# Care coordination for children with special health care needs: a cohort study

**DOI:** 10.1186/s13052-017-0342-3

**Published:** 2017-02-03

**Authors:** Elisa Zanello, Simona Calugi, Lee M. Sanders, Jacopo Lenzi, Giacomo Faldella, Paola Rucci, Maria Pia Fantini

**Affiliations:** 1 Department of Biomedical and Neuromotor Sciences, Division of Hygiene and Biostatistics, Alma Mater Studiorum University of Bologna, Via San Giacomo 12, Bologna, 40126 Italy; 2 Division of General Pediatrics, Senior Faculty, Center for Policy, Outcomes and Prevention (CPOP), Stanford University, 117 Encina Commons, Stanford, CA 94305-6019 USA; 3 Department of Medical and Surgical Sciences, Neonatology and Neonatal Intensive Care Unit, S.Orsola Malpighi Hospital, University of Bologna, Via Massarenti 11, Bologna, 40138 Italy

**Keywords:** Children with special healthcare needs, Pediatric primary care, Family pediatrician, Care coordination, Assessment

## Abstract

**Background:**

Care coordination is widely recognized as a key element of care for patients with chronic and complex medical conditions and their families. In care for children with special health care needs the Family Pediatrician (FP) plays a central role as care coordinator. This study aims to evaluate the FPs’ activities of care coordination for children with special health care needs in the pediatric primary care setting, using an on-line measurement tool.

**Methods:**

Within the prospective cohort study SpeNK (Special Needs Kids), newborns and children with special health care needs were recruited at discharge from three hospital facilities in Bologna province, from October 1st 2012 to September 30th 2014. Their FPs were invited to complete a questionnaire (SpeNK-FP) at each encounter for the patient during a 9-month period after hospital discharge. SpeNK-FP was developed by adapting the Care Coordination Measurement Tool (CCMT©) developed by Antonelli et al., to the Italian organizational context. The outcome of interest, derived from the questionnaire, is inappropriate use of services.

**Results:**

Forty FPs completed assessments for 49 children at each of 382 clinical encounters. The majority of children (71.4%) had special health care needs, without complicating social issues. FPs reported “no need for care coordination” in 50.8% of the encounters and 41.1% of records about patient needs requiring care coordination. The most common activity implemented to meet children’s needs was telephone contact with a medical provider. According to FPs, 80% of encounters prevented inappropriate services use. In multivariate regression, pediatric-specialist contact (telephone or in person) was associated with reduced odds of physician report of preventable hospitalization (OR = 0.06, 95% CI 0.01–0.42, *p* = 0.005).

**Conclusions:**

The study shows the potential for FPs in Italy to serve as care coordinators and facilitate the implementation of integrated care pathways for children with special health care needs.

**Electronic supplementary material:**

The online version of this article (doi:10.1186/s13052-017-0342-3) contains supplementary material, which is available to authorized users.

## Background

Care coordination has been widely recognized as an important process of organization of patient care activities to facilitate the appropriate delivery of health care services and to achieve a high-quality, high-value, patient-centered health care system [1]. The goal of care coordination is to support patients and their families requiring health care in their interaction with an increasingly complex health care system.

In the context of pediatric health care, care coordination has been defined as “a process that links children and youth with special health care needs and their families with appropriate services and resources in a coordinated effort to achieve good health” [2]. Children with special health care needs can be defined as those who “*have, or are at an increased risk for, a chronic physical, developmental, behavioral, or emotional conditions and who also require health and related services of a type or amount beyond that generally required by children*” [3]. This definition refers to a “heterogeneous population” with a variety of “diagnoses and functional limitations”, sharing in common a “high need for services” [4]. Care coordination for this population is associated with lower odds of unmet specialty care needs [5].

A key role in care coordination for children with special health care needs may be played by the primary care provider [6, 7]. However, some studies underlined the scant involvement of primary care providers in managing care of children with chronic conditions [8, 9].

In Italy, family pediatricians (FPs) are trained specialists providing primary care for children up to 16 years of age in ambulatory and home settings and coordination of care for patients with chronic conditions [10]. FPs are in charge of assessing patients’ needs, ordering diagnostic procedures, prescribing drugs, and referring patients to specialists and hospitals [11].

The Italian Collective Agreement (July 2010) governs the relationship of the National Health Service with FPs and includes, among the general objectives, the construction of an integrated network of services for children with special health care needs and for the government of health care and social pathways.

Nevertheless, research about the implementation of care coordination for children with special health care needs, and specifically about the role of FP as care coordinator is limited.

The aim of this study is to describe the care coordination activities performed by FPs in the primary care setting for children with special health care needs. The study is embedded in a government-funded program, called Special Needs Kids (SpeNK) Research Project, in Emilia-Romagna, a Northern Italy Region. The SpeNK Project was designed to improve the hospital-discharge procedures and integrated clinical pathways for children with complex or chronic health conditions. Special health care needs of the children and the family’s perspective on continuity of care and the role of FP were also studied.

## Methods

### Study design

In order to assess the activities that FPs performed for the coordination of care of children with special health care needs after their hospital discharge, in a 9-months follow-up period, the study involved FPs who were in charge of children recruited to SpeNK Project.

Children and families were recruited for SpeNK Project at hospital discharge of the child from the participating units of St. Orsola Malpighi University Hospital of Bologna and Local Health Authorities of Bologna and Imola [12, 13], Children recruitment was conducted from October 1st 2012 to September 30th 2014 on incident cases meeting the following inclusion criteria: age from 0 to 16 years, residence in Bologna province, and the presence of at least one of the following conditions:

- Birth weight <1000 g;
- Complex and/or chronic health conditions defined as: need for technological assistance, acute neurological deficit, severe endocrinopathy, complex genetic malformative pathology;
- Children with oncological diseases who need palliative care or particular community care;
- Newborns with mothers in contact with mental health services or drug addiction.

Only first-ever hospitalizations for the condition of interest were included. Written informed consent was obtained at recruitment from each parent to collect clinical data on children during the follow-up period (9 months from hospital discharge).

The involvement of FPs was arranged by the SpeNK Team in coordination with the Departments of Primary Care of the Local Health Authorities of Bologna and Imola and the local branches of two FPs’ trade unions (FIMP [*Federazione Italiana Medici Pediatri*] and C.I.Pe. [*Confederazione Italiana Pediatri*]). An agreement was reached and FPs who participated on a voluntary basis in the study received a small participation fee.

The role of FPs in the care management of chronic conditions was first outlined in the National Collective Agreement of 2000 (DPR 272/2000). More recently, the legislative decree no. 158/2012 (http://www.gazzettaufficiale.it/eli/id/2012/11/10/12A11988/sg) established that Regions define the organization of local primary care services by promoting integration with social and hospital services. This is done through monoprofessional organizational arrangements, called local functional aggregations, that share objectives, care pathways and clinical guidelines, as well as multiprofessional organizational arrangements, named complex primary care units, that deliver care through coordination and integration of physicians with other health care professionals affiliated with the National Health Service. In Emilia-Romagna Region, the 2010 FPs’ Regional Collective Agreement, still in force, establishes economic incentives to promote the care of children with chronic health conditions; however, FPs are not required to record their activities and to date there is no tool to monitor FP care delivered for chronic conditions.

### Measurement instrument

The care coordination measurement instrument (SpeNK-FP) was developed adapting the CCMT^©^ by Antonelli et al. [14] to the Italian organizational context. It was prepared in an online version to be completed by the FP at each encounter regarding the child with special health care needs. The “Encounter” was defined as “any activity performed by the FP for the patient”, involving the child or the family and including visit at the clinic, phone contact, etc.

SpeNK-FP included an “identity record” and an “encounter record”. The identity record collected the personal data of the patient (name, gender, birth date, etc.). The encounter record included the date of the encounter and eight items aiming to collect information about the activity performed by the FP. The first item concerns the patient’s clinical and social complexity on a three levels scale: children with mainly social needs (Level 1), children with mainly health needs (Level 2), children with both health and social needs (Level 3). The second item inquires the request(s) or problem(s) of the patient for which the encounter took place (e.g. make appointment with the FP, referral to a subspecialist). Item 3 investigates the need(s) for care coordination that emerged in the encounter (e.g. make appointments with other specialists). Item 4 investigates which activities were carried out by the FP (e.g. contacts with family or with hospital) to fulfill patient’s needs emerged in the encounter. Item 5 examines the involvement of any other professional(s) in the care coordination activity. Item 6 inquires the time spent for care coordination. The two final items inquire the FP’s appraisal about the outcomes occurred and prevented with the care coordination activity.

An additional file presents the instrument in its original form (Italian language) [see Additional file 1].

### Training and data collection

The SpeNK Team planned and provided a 1-day training program about the study design and data collection procedures in two separated sessions for the FPs of Imola and Bologna respectively. FPs were asked to record each encounter during the follow-up period.

A member of the SpeNK Team contacted the FP to alert the beginning of follow-up for each subject after hospital discharge, to remind aims and procedures of the study, and to provide the login credentials and the instructions to use the SpeNK-FP on-line form. During follow-up, the same Team member maintained telephone contacts with FPs to provide technical support and to alert the end of the follow-up period for each enrolled child. Ethical approval for the study was obtained from the University Hospital and Local Health Authorities’ Ethics Committees (protocol No. 64/2012/O/OSS of Bologna University Hospital, protocol No. 12046–SpenK of Bologna Local Health Authority, protocol No. 140/2012/O/OSS of Imola Local Health Authority).

### Statistical analysis

Data were summarized using frequencies, percentages, mean, standard deviation and range. A logistic multilevel analysis was carried out in order to investigate the relationship between each prevented outcome and each activity undertaken by the FP, to ascertain what coordination activities were more preventive and, if so, on what outcomes. This approach was chosen to take into account the hierarchical nature of the data (encounters nested into patients), which violates the assumption of independence required by regression models.

## Results

A total of 61 FPs who were in charge of 82 children with special health care needs recruited to SpeNK study were contacted and invited to participate in the study. Of these, 40 FPs (65.6%) completed the SpeNK-FP for a total of 49 (59.7%) children.

Thirty-one FPs were female (87.5%) , with a mean age of 55.3 years (SD = 5.6, range = 37–66). Their practices were located mainly in areas with middle and high urbanization (16 and 18 respectively).

The 49 children were 53.1% male (*n* = 26), with a mean age of 5.8 months (SD = 11.8, range = 0–76). 21 (42.9%) were preterm infants with birth weight < 1000 g, 7 (14.3%) had complex genetic or malformative pathologies, 5 (10.2%) had a diagnosis of prematurity with other conditions, 5 (10.2%) of encephalopathy or neuropathy, 7 (14.3%) of other medical conditions, and 4 (8.2%) had social problems. Assessed by levels of complexity, three children (6.1%, with a total of 19 encounters) were assessed at Level 1 (mainly social concerns), 35 children (71.4%, with a total of 290 encounters) at Level 2 (mainly health concerns), and 11 children (22.5%, with a total of 73 encounters) at Level 3 (both health and social concerns).

The total number of encounters studied was 382, with a mean of 12.7 (SD = 6.9) clinical encounters per child (median = 12.5, range 1–28). Overall, each FP completed questionnaires for a mean of 17.0 (SD = 10.3, median = 15, range = 1–36) encounters. Time spent per encounter was less than 5 min in 5.3% of encounters of children at Level 1, 13.8% at Level 2, and 25% at Level 3. On the other hand, time spent per encounter was greater than 30 min in 5.3% of encounters of children at Level 1, 29.2% at Level 2, and 19.4% at Level 3.

Table 1 shows the focus of encounters. In 29.6% of cases, the focus included growth and nutrition issues, in 24.2% the request of a FP’s visit, and in 21.1% developmental and behavioral issues.

**Table 1:** Focus of encounters with Family Pediatricians

Focus of encounters	No. recorded, n (%)^a^
Convene meeting with Family Pediatrician	206 (24.2%)
Medicines prescriptions	79 (9.3%)
Prescriptions of laboratory examinations	29 (3.4%)
Prescriptions of laboratory tests	3 (0.4%)
Need for prosthesis/devices	3 (0.4%)
Growth and nutrition	252 (29.6%)
Referral management	32 (3.8%)
Developmental and behavioral	179 (21.1%)
Educational and school	3 (0.4%)
Mental health	0
Social services (i.e. housing, food, clothing. etc.)	14 (1.6%)
Integrated Home Care (i.e. *ADI*)	21 (2.5%)
Legal and Judicial	1 (0.1%)
Other	28 (3.3%)

^a^Total 850 recorded focuses for 376 encounters

Table 2 shows patient’s needs requiring care coordination. For more than 40% (*n* = 150) of patients’ needs, FPs reported “no need for care coordination”, referring to 50.8% (*n* = 150) of the encounters with at least one need recorded (*n* = 295). Care coordination was required mainly for follow-up referrals (27.1%), laboratory examinations (9.6%) and tests (4.4%), and prescriptions (7.9%).

**Table 2:** Patient’s needs requiring care coordination emerged during the encounter with the Family Pediatrician

Patient’s needs requiring care coordination	No. recorded, n (%)^a^
Follow-Up Referrals	99 (27.1%)
Order Prescriptions	29 (7.9%)
Order Supplies	7 (1.9%)
Order Services	15 (4.1%)
Order Laboratory Examinations	35 (9.6%)
Order Laboratory Tests	16 (4.4%)
Coordination Services (schools, agencies, payers, etc.)	4 (1.1%)
Reconcile Discrepancies	9 (2.5%)
None	150 (41.1%)

^a^Total 364 recorded needs for 295 encounters

FPs recorded 468 activities to fulfill patient’s needs for 376 encounters. More than half of these activities included contacts with health care professionals and services: 71 (15.3%) contacts with hospital or clinic, 71 (15.1%) meetings or case reviews, 49 (10.4%) contacts with specialists, and 33 (7%) contacts with social and health services. About one-third of these activities (*n* = 158, 33.6%) included contacts with parent/family. About one-third of contacts (*n* = 128, 34%) were face-to-face meeting, and 113 (30%) were by telephone. Only 5 (1.3%) contacts took place by e-mail.

FPs entered 422 records about staff involved in care coordination, other than FP, concerning 376 encounters. In more than half of cases, no one was involved (*n* = 219, 51.9%), in 22% of cases other physicians (*n* = 93), and in the remaining 26.1% of cases other staff, i.e. nurse(s) (*n* = 36, 8.5%), social worker(s) (*n* = 38, 9.0%), administrative staff (*n* = 13, 3.1%), other (*n* = 23, 5.5%).

Table 3 shows the subjective assessment of FPs about the outcomes prevented because of their care coordination activity. Nearly 80% of records reported that encounters prevented an inappropriate use of services (i.e. visits to the emergency room, hospital admissions, subspecialist visits, and visits to the Pediatric Office).

**Table 3:** Outcomes prevented because of Family Pediatricians’ care coordination activity

Prevented outcomes	No. recorded, n (%)^a^
Emergency Room visit	113 (18.3%)
Subspecialist visit	144 (23.4%)
Hospitalization	85 (13.8%)
Visit to Pediatric Office/Clinic	150 (24.4%)
Lab/X-ray	63 (10.2%)
Drugs	52 (8.4%)
Other	9 (1.5%)

^a^Total 616 recorded outcomes for 286 encounters

Multivariate analysis revealed that contacts with specialists were significantly and strongly associated with physician report of prevented hospitalizations (odds ratio [OR] = 0.06, 95% CI 0.01–0.42 , *p* = 0.005). This result is supported by the fact that 13 out the 19 patients whose FPs had contacts with specialists had a prevented hospitalization (68%), while among the 30 patients whose FPs had no contacts with specialists only nine experienced this prevented outcome (30.0%). There were non-significant associations between case-review activities and prevented hospitalizations (OR = 0.38, 95% CI 0.12–1.22, *p* = 0.104) and prevented Emergency Department visits (OR = 0.49, 95% CI 0.18–1.32, *p* = 0.160). None of the other care-coordination activities were associated with the physician report of prevented hospitalization or Emergency Department use.

An additional file provides the complete dataset of the encounters [see Additional file 2].

## Discussion

This study aimed to assess the care coordination activities performed by the FPs for children and newborns with special health care needs in the pediatric primary care setting. To achieve this aim, we involved FPs of children recruited to the SpeNK Project during 9 months after hospital discharge.

Our first finding is about the scant participation of FPs. In fact, only 65.6% of recruited FPs recorded contacts. In the Italian Health care System, differently from other countries (e.g. France, Germany, U.S.A.) where FPs are paid “fee for service”, FPs and are not required to record their activities, since they are mostly remunerated on a capitation base formula. This could partially explain the low compliance of our FPs.

Another explanation is that FPs perceive the time spent in documenting their activities as time diverted from clinical care. This is consistent with the findings of a study conducted in U.S. by Agrawal et al. [15] in large sample of FPs in Illinois, in which issues related to time spent, coding and billing are perceived as significant barriers to care children and youth with special health care needs. This evidence suggests that, in general, documenting FPs’ care coordination activities is complex and, as suggested by Antonelli, far away from being “measurable, auditable, and amenable to continuous quality improvement” [14]. Moreover, the low compliance in the use of the online form could be explained by the age and gender of FPs involved. In fact, older people (55–74 years, representing 67.5% of our sample) as well as women (87.5%) tend to have lower digital competence (https://ec.europa.eu/digital-agenda/sites/digital-agenda/files/KKAH12001ENN-chap5-PDFWEB-5.pdf).

The second finding is about the health care needs across the complexity levels of patients. Encounters were recorded by FPs across three patient complexity levels, with patients with mainly health needs (Level 2) representing the majority of the sample and receiving the majority of the activities and time spent. This result is in contrast with the findings of the study by Antonelli et al. [14], who argue that the presentation of an acute, family-based social stressor demands the provision of significant care coordination services. We wonder if, in our study population, the presence of social and familial problems adversely affects the access to appropriate services, as suggested by the literature about the barriers to health services use and access in vulnerable groups [16–18].

The third and main finding regards the focus of encounters. Our results indicate that the FP’s role is mainly clinical, referring to growth/nutrition issues, visits and development/behavioral matters, which represent focuses similar to primary care for children without special health care needs. Similarly, our results indicate that the FP’s role is to a lesser extent of care coordination, since FPs identified no need for care coordination in 40% of recorded patients’ needs. Moreover, according to FPs, the patients’ needs requiring care coordination concern mainly referrals, prescriptions and labs. This finding is consistent with a qualitative study by Zanello et al. [12], in which parents reported mixed perceptions and experiences about FPs, related to their centrality vs. marginality in the activation and coordination of the health care network. Of note, although some Italian regions such as Emilia-Romagna and Tuscany have started the implementation of the “Chronic Care Model” in which care coordinators and case managers play a pivotal role, these health professionals are still uncommon in the services that are in charge of children with special health care needs. Another explanation of our finding might be the lack of specific training and preparation about care coordination tasks during the academic education and professional life [6, 19, 20].

The last finding indicated that 80% of encounters, according to the FPs, prevented an inappropriate use of services (i.e. visits to the emergency room, hospital admissions, subspecialist visits, and visits to the Pediatric Office). This result is consistent with the results of the study by Antonelli et al. [14] in which care coordination encounters prevented unnecessary service use. We also found that, among all care coordination activities performed by the FPs, contacts with specialists were significantly associated with prevented hospitalizations. A study by Christakis et al. [21] similarly found a decreased risk of Emergency Department use and hospitalization in children having consistent contacts with a primary care provider. These findings fit with the working definition developed by McDonald et al. [22] stating that “organizing care involves the marshalling of personnel and other resources needed to carry out all required patient care activities and is often managed by the exchange of information among participants responsible for different aspects of care”.

Our study has two main limitations. First, the small sample size and the low compliance of FPs do not allow to generalize our findings. Second, the poor quality of data recorded about time spent does not permit to calculate the real amount of time spent for specific care coordination activities.

Nevertheless, our study is the first in Italy examining and measuring with a standardized instrument the activities performed by FPs to care for children with special health care needs and to coordinate the care provided by multiple services and professionals.

Some implications for practice, policy and research emerged from our study. Our findings suggest the need to provide greater structural and process support for FPs’ care coordination activities, in particular about referrals, labs and prescriptions. Health care policies aiming to increase the involvement of non-physician staff in care coordination activities could help the pediatric primary care services and providers meet needs of children with special health care needs and their families. Similar studies in other countries are warranted to compare the care coordination activities performed by primary care providers for children with special health care needs in different health care systems.

## Conclusions

Assessing the FP’s role in care coordination for children with special health care needs is a challenge for health care systems. Even when closely monitored, according to these Italian primary care providers, the needs of children with special health care needs require coordination of care only in 50% of encounters. In order to meet needs of these children and their families, a more supportive system of outpatient primary care may be necessary, leveraging the skills and experience, perhaps, of non-physician staff and community members.

## Additional files

Additional file 1: The SpeNK-FP instrument in its original form (Italian language). “*Scheda per l’attività di coordinamento del PLS”*. (PDF 590 kb)
Additional file 2: The dataset of the encounters and the codebook. “*SpeNK-FP records about activity performed by FPs*”. (XLS 255 kb)
