# Supplementary material for: Care coordination for children with special health care needs: a cohort study
Source: Ital J Pediatr. 2017 Feb 3;43:18. doi: 10.1186/s13052-017-0342-3 (PMC5347827; doi:10.1186/s13052-017-0342-3)
Supplement: Additional file 1: — The SpeNK-FP instrument in its original form (Italian language). “Scheda per l’attività di coordinamento del PLS”. (PDF 590 kb) [file 13052_2017_342_MOESM1_ESM.pdf]

SCHEDA PER L'ATTIVITÀ DI COORDINAMENTO DEL PLS

(Adattamento del *Medical Home Care Coordination Measurement Tool*© sviluppato da Antonelli et al., 2008)

SCHEDA ANAGRAFICA

Cognome: ____ *(campo obbligatorio)*

Nome: ____ *(campo obbligatorio)*

Data di nascita: |___|___|/|___|___|/|___|___|___|___| *(campo obbligatorio)*

Cod. Fiscale: |___|___|___| |___|___|___| |___|___|___|___|___| |___|___|___|___|___| *(campo obbligatorio)*

Cod. Esenzione: |___|___|___| |___|___|___|___|___|
(Cod. ICD-9-CM)

(tutti i campi sono *obbligatori*)

SCHEDA CONTATTO

Data contatto: |___|___|/|___|___|/|___|___|___|___| *(campo obbligatorio)*

LIVELLO COMPLESSITÀ DEL PAZIENTE

Definire, sulla base dei dati clinici in proprio possesso, il livello di complessità assistenziale del proprio assistito (indicane SOLO UNO):

☐ Bambini senza particolari bisogni di assistenza sanitaria, con questioni familiari o problemi sociali complicanti

☐ Bambini con particolari bisogni di assistenza sanitaria, senza questioni familiari o problemi sociali complicanti

☐ Bambini con particolari bisogni di assistenza sanitaria, con questioni familiari o problemi sociali complicanti

MOTIVO DEL CONTATTO

Specificare per quale delle seguenti problematiche/richieste del paziente è avvenuto il contatto (possibilità di scelta multipla):

☐ Richiesta visita al PLS

☐ Prescrizione farmaci

☐ Prescrizione esami strumentali

☐ Prescrizione esami di laboratorio

☐ Necessità protesi/ausili

☐ Crescita/nutrizione

☐ Invio ad uno specialista

☐ Sviluppo psicomotorio/comportamento

☐ Educazione/scolarizzazione

☐ Ritardo mentale

☐ Servizi sociali *(alloggio, alimentazione, abbigliamento, assicurazione, trasporti)*

☐ Richiesta ADI

☐ Questioni legali/giudiziarie

☐ Altro

BISOGNI CHE RICHIEDONO ATTIVITÀ DI COORDINAMENTO

Indicare quali dei seguenti bisogni del paziente, emersi durante il contatto, richiedono attività di assistenza coordinata (possibilità di scelta multipla):

☐ Valutare ulteriori rinvii specialistici

☐ Richiedere farmaci

☐ Richiedere attrezzature sanitarie

☐ Richiedere servizi

☐ Richiedere esami strumentali

☐ Richiedere esami di laboratorio

☐ Servizi di coordinazione *(scuole, associazioni, assicurazione)*

☐ Risoluzione incomprensioni e mancate comunicazioni

☐ Nessuno

ATTIVITÀ MESSE IN PRATICA

Specificare le attività da mettere in pratica per soddisfare i bisogni del paziente emersi durante il contatto (possibilità di scelta multipla):

☐ Contatti con genitori/famiglia (Se "SI", marcare una modalità)

☐ Telefono ☐ Incontri ☐ Lettera ☐ E-mail ☐ Fax

☐ Contatti con scuola (Se "SI", marcare una modalità)

☐ Telefono ☐ Incontri ☐ Lettera ☐ E-mail ☐ Fax

☐ Contatti con Servizi socio-sanitari (Pediatria di Comunità, Servizi Sociali, …) (Se "SI", marcare una modalità)

☐ Telefono ☐ Incontri ☐ Lettera ☐ E-mail ☐ Fax

☐ Contatti con ospedale/clinica (Se "SI", marcare una modalità)

☐ Telefono ☐ Incontri ☐ Lettera ☐ E-mail ☐ Fax

☐ Contatti con assicurazione (Se "SI", marcare una modalità)

☐ Telefono ☐ Incontri ☐ Lettera ☐ E-mail ☐ Fax

☐ Contatti con educatore professionale (Se "SI", marcare una modalità)

☐ Telefono ☐ Incontri ☐ Lettera ☐ E-mail ☐ Fax

☐ Contatti con farmacia (Se "SI", marcare una modalità)

☐ Telefono ☐ Incontri ☐ Lettera ☐ E-mail ☐ Fax

☐ Contatti con associazioni (Se "SI", marcare una modalità)

☐ Telefono ☐ Incontri ☐ Lettera ☐ E-mail ☐ Fax

☐ Contatti con personale dell'ADI (Se "SI", marcare una modalità)

☐ Telefono ☐ Incontri ☐ Lettera ☐ E-mail ☐ Fax

☐ Contatti con specialisti (Se "SI", marcare una modalità)

☐ Telefono ☐ Incontri ☐ Lettera ☐ E-mail ☐ Fax

☐ Contatti con altra figura (Se "SI", marcare una modalità)

☐ Telefono ☐ Incontri ☐ Lettera ☐ E-mail ☐ Fax

☐ Comunicazioni scritte (Se "SI", marcare una modalità)

☐ Rilascio certificati/moduli per scuole e tempo libero
☐ Rilascio referti/risultati indagini di laboratorio e strumentali
☐ Nessuna

☐ Notifiche/relazioni alle Autorità sanitarie (notifica malattie infettive, …)

☐ Revisione/aggiornamento documentazione paziente

☐ Audit clinico

☐ Sviluppare/modificare il piano di assistenza individuale (PAI)

☐ Incontri/conferenze sul caso

STAFF COINVOLTO NELL'ATTIVITÀ DI COORDINAMENTO

Nella programmazione delle attività di coordinamento specificare quali figure professionali del tuo staff sono coinvolte(possibilità di scelta multipla):

☐ Nessuno

☐ Altri medici

☐ Infermieri professionali

☐ Operatori sociali

☐ Personale amministrativo

☐ Altro

TEMPO DEDICATO ALLE ATTIVITÀ DI COORDINAMENTO

Quantifica il tempo dedicato alla risoluzione del problema e alle conseguenti attività di coordinamento necessarie(indicane SOLO UNO):

☐ Meno di 5 minuti

☐ Da 5 a 9 minuti

☐ Da 10 a 19 minuti

☐ Da 20 a 29 minuti

☐ Da 30 a 39 minuti

☐ Da 40 a 49 minuti

☐ = o >50 minuti (prego annotare i minuti effettivi, se il tempo è >50') |___|___|___|minuti

ESITI DELL'ATTIVITÀ DI COORDINAMENTO

Grazie alla presente valutazione, secondo la tua opinione, non è stato necessario per il paziente il ricorso a: (possibilità di scelta multipla):

☐ Visita al Pronto Soccorso

☐ Ulteriore visita specialistica

☐ Ospedalizzazione

☐ Visita alla clinica pediatrica

☐ Indagini di laboratorio/indagini strumentali

☐ Farmaci

☐ Altro (***specificare)*** ___________________________

Come risultato del presente contatto, si è verificato il seguente esito (possibilità di scelta multipla):

☐ Aver consigliato la famiglia/il paziente in merito alla gestione domiciliare

☐ Invio al Pronto Soccorso

☐ Invio ad ulteriore specialista

☐ Invio per ricovero

☐ Invio per visita alla clinica pediatrica

☐ Invio per indagini di laboratorio/indagini strumentali

☐ Utilizzo farmaci

☐ Aver richiesto attrezzature, pannolini, trasporti

☐ Aver risolto questioni legate a dati mancanti, mancate comunicazioni, problemi burocratici

☐ Aver riesaminato dati laboratorio, resoconti degli specialisti, programmi di educazione individuale,ecc.

☐ Attività di sostegno per la famiglia/paziente

☐ Aver provveduto ai bisogni immediati, alle domande e alle preoccupazioni della famiglia

☐ Problemi non risolti

☐ Altro

NOTE

____
____
____
____
____
____
____
____
____
____
____
____
____
____
____
____
____
____
____
____
